# Lithium ameliorates autistic-like behaviors induced by neonatal isolation in rats

**DOI:** 10.3389/fnbeh.2014.00234

**Published:** 2014-06-26

**Authors:** Xiaoyan Wu, Yanrui Bai, Tao Tan, Hongjie Li, Shuting Xia, Xinxia Chang, Zikai Zhou, Weihui Zhou, Tingyu Li, Yu Tian Wang, Zhifang Dong

**Affiliations:** ^1^Ministry of Education Key Laboratory of Child Development and Disorders, Children’s Hospital of Chongqing Medical UniversityChongqing, China; ^2^Chongqing Key Laboratory of Translational Medical Research in Cognitive Development and Learning and Memory Disorders, Children’s Hospital of Chongqing Medical UniversityChongqing, China; ^3^Ministry of Education Key Laboratory of Developmental Genes and Human Disease, Institute of Life Sciences, Southeast UniversityNanjing, China; ^4^Brain Research Centre, University of British ColumbiaVancouver, BC, Canada

**Keywords:** neonatal isolation, autism spectrum disorder, social deficit, neurogenesis, spontaneous inhibitory postsynaptic current

## Abstract

Neonatal isolation is a widely accepted model to study the long-term behavioral changes produced by the early life events. However, it remains unknown whether neonatal isolation can induce autistic-like behaviors, and if so, whether pharmacological treatment can overcome it. Here, we reported that newborn rats subjected to individual isolations from their mother and nest for 1 h per day from postnatal days 1–9 displayed apparent autistic-like symptoms including social deficits, excessive repetitive self-grooming behavior, and increased anxiety- and depressive-like behaviors tested in young adult (postnatal days 42–56) compared to normal reared controls. Furthermore, these behavioral changes were accompanied by impaired adult hippocampal neurogenesis and reduced the ratio of excitatory/inhibitory synaptic transmissions, as reflected by an increase in spontaneous inhibitory postsynaptic current (sIPSC) and normal spontaneous excitatory postsynaptic current (sEPSC) in the hippocampal CA1 pyramidal neuron. More importantly, chronic administration of lithium, a clinically used mood stabilizer, completely overcame neonatal isolation-induced autistic-like behaviors, and restored adult hippocampal neurogenesis as well as the balance between excitatory and inhibitory activities to physiological levels. These findings indicate that neonatal isolation may produce autistic-like behaviors, and lithium may be a potential therapeutic agent against autism spectrum disorders (ASD) during development.

## Introduction

Autism spectrum disorder (ASD) is a highly prevalent neurodevelopmental disorder (Mulvihill et al., 2009), which is characterized by impaired social interactions and enhanced repetitive or stereotyped behavior, as well as marked inflexibility to environmental contingency changes (American Psychiatric Association, 1994; Arndt et al., 2005; DiCicco-Bloom et al., 2006). The prevalence of autism is about 3–6 per 1000 people (Rutter, 2005). The number of new autism diagnoses has increased dramatically since the 1980s, in large part due to better ascertainment and a broadening of the diagnostic concept. According to a recent report from the Centers for Disease Control and Prevention (CDC), around 20 per 1000 children aged 6–17 in the United States are diagnosed with ASD in 2011–2012 (Blumberg et al., 2013). The cause of autism appears to be primarily genetic, with an estimated heritability of over 90% obtained from epidemiological studies of autistic twins (Bailey et al., 1995). Therefore, most previous studies have focused on the role of congenital genetic factors in autism (Abrahams and Geschwind, 2008; State and Levitt, 2011; Devlin and Scherer, 2012). Correlations between acquired factors and autism occurrence have not been extensively investigated, although there is a growing body of evidence to show that valproic acid exposure, around the critical time period of neural tube closure, leads to autistic-like anatomical and behavioral phenotypes in the offspring in human and in animal models (Christianson et al., 1994; Schneider and Przewlocki, 2005; Christensen et al., 2013).

Neonatal physiology and development are regulated by the ongoing mother-infant interactions. Daily maternal separation during the early postnatal period appears to be stressful to the pup and leads to changes in many adult attentional, affective and emotional behaviors, as well as the impairment of adult neurogenesis (Kuhn et al., 1990; Rhees et al., 2001; Francis et al., 2002; Plotsky et al., 2005; Rizzi et al., 2007; Kikusui et al., 2009; Raceková et al., 2009). Furthermore, studies from animal models show that neonatal isolation significantly impairs learning and memory (Kosten et al., 2007; Marco et al., 2013), and increases vulnerability of the adolescent or adult to drug abuse (Kehoe et al., 1996; Kosten et al., 2006). Nonetheless, contradictory results challenge these findings. For example, recent studies show that neonatal isolation enhances hippocampal LTP and object location memory (Kehoe et al., 1995; Makena et al., 2012). These discrepancies may be accounted for by developmental stages of the subjects, species variations and different behavioral tasks used in these studies. To date, effects of neonatal isolation on autistic-like behaviors such as social interactions and repetitive self-grooming behaviors are largely unknown, although a recent report show that ovariectomized female mice subjected to maternal separation display social deficit (Tsuda and Ogawa, 2012). Thus, the present study investigated the effects of neonatal isolation on autistic-like behaviors including social behavior, repetitive self-grooming behavior and anxiety/depressive-like behaviors in both male and female rats.

As a mood stabilizer, lithium remains a major choice in both acute and long-term clinical management of manic-depressive disorder (Manji et al., 2000). In addition, previous studies show that lithium treatment displays obvious benefits in a variety of animal models relevant to human neurodegenerative diseases including Alzheimer disease (De Ferrari et al., 2003) and Huntington’s disease (Senatorov et al., 2004), and neurodevelopmental disorders such as fragile X (Guo et al., 2011) and Down syndrome (Bianchi et al., 2010; Contestabile et al., 2013). More recent studies report that chronic lithium treatment has a neuroprotective effect on hypoxic-ischemic brain injury in the neonatal rat (Li et al., 2011; Shin et al., 2012). The potential mechanisms underlying some of the beneficial effects of lithium remain not fully understood, but it may involve reducing apoptotic neuronal loss, stimulating neural precursor cell (NPC) proliferation and cell differentiation into mature neurons, as well as increasing the level of neurotrophic factors (Chen et al., 2000; Son et al., 2003; Senatorov et al., 2004; Contestabile et al., 2013). However, it is unclear whether lithium can exert some beneficial effects on abnormal behavioral changes caused by neonatal isolation.

In the present study, we investigated autistic-like behavioral changes in rats subjected to neonatal isolation from postnatal days 1–9 (PND 1–9). We selected three behavioral patterns corresponding to those frequently observed in children with autism for examination under laboratory conditions: (1) social interaction deficit, a hallmark feature of autism (American Psychiatric Association, 1994); (2) repetitive self-grooming behavior, another core symptom of autism (Lewis et al., 2007; Silverman et al., 2010; Bishop and Lahvis, 2011); and (3) anxiety- and depression-related behaviors, which are frequently observed in autistic patients (Mazefsky et al., 2010; Strang et al., 2012). We found that when tested in young adults, neonatal isolation led to social deficit, excessive self-grooming and increased anxiety/depressive-like behaviors. In addition, neonatal isolation significantly decreased adult neurogenesis in the dentate gyrus (DG) and enhanced basal inhibitory synaptic transmission in the hippocampal CA1 pyramid neurons. More importantly, we found that chronic treatment with lithium, a mood stabilizer, can significantly ameliorate these abnormal changes.

## Materials and methods

### Animals

All procedures were performed in accordance with Chongqing Science and Technology Commission guidelines for animal research and approved by the Chongqing Medical University Animal Care Committee. Female and male Sprague–Dawley rats were mated (obtained from Chongqing Medical University Animal Care Centre) in the laboratory colony of Children’s Hospital of Chongqing Medical University, and the offspring of these matings served as subjects. Pregnant females were housed individually in plastic cages in the temperature-controlled (21°C) colony room on a 12/12 h light/dark cycle (8 am–8 pm). Food and water were available *ad libitum*. Within 24 h of birth, all litters were culled to 10 pups with a goal of balancing the number of males and females equally. Culled litters were randomly assigned as either neonatal isolation (Iso) or normal reared control (Ctr). All experiments were carried out in young adult rats from PND 42 to PND 56.

### Reagents and antibodies

Lithium chloride (LiCl) and bromodeoxyuridine (BrdU) were purchased from Sigma-Aldrich, and dissolved in 0.9% sterile saline at required concentrations.

Mouse Anti-BrdU monoclonal antibody was obtained from Sigma-Aldrich. Rabbit anti-NeuN monoclonal antibody was obtained from Millipore. Rabbit anti-GFAP monoclonal and rabbit anti-doublecortin (DCX) polyclonal antibodies were obtained from Abcam. Complete protease inhibitor cocktail tablets and phosphatase inhibitor cocktail tablets were purchased from Roche Applied Science.

### Neonatal isolation

Litters born before 5:00 pm were considered born on PND 0. From PND 1 to PND 9, one half of pups were designated for isolation. During the isolation procedure, each pup was placed approximately 15 cm apart in an individual round plastic chamber (9 cm in diameter, 8 cm in deep) with no bedding for 1 h, and the chamber temperature was kept at 30°C. Isolations were carried out between 9 am and 12 am each day. Immediately after daily isolation, pups were assigned to two subgroups, which were subjected to LiCl (2 mmol/kg, i.p.) or same volume of saline injection, and then pups were transferred back to the original nest with their dam. After 9 days in isolation, pups remained with their mothers continuously until weaning on PND 21, and they were housed in same-sex pairs of the same treatment condition in the same colony facility.

### Social interaction

The social interaction test was performed in a three-chambered apparatus, which was made from white Plexiglas and contained three chambers with the same dimensions (length × width × height = 20 × 40 × 20 cm for each). An identical cage was used to enclose a stranger rat or object in each side chamber, and the central chamber was empty. Animal used as “stranger” was a SD rat of the same gender with the same age and no previous contact with the test rats. Test rats were individually acclimated for 5 min into the empty apparatus on the day before the experiment. On the test day, rats were individually tested for 10 min, and the entries and time spent in each chamber was monitored by ANY-Maze Video Tracking System (Stoelting Co.). Chambers were cleaned with 70% ethanol and water between tests.

### Self-grooming behavior

Animals were acclimated for 5 min in an open-field arena (60 × 60 cm). Twenty-four hours after habituation, animals were video recorded for 15 min and later scored for self-grooming behavior. Self-grooming was defined as time spent licking paws, washing the nose, face or scratching fur with any foot. The box was cleaned with 70% ethanol and water between tests.

### Elevated plus maze

Rats were placed in the centre of a plus maze (each arm 50 cm) that was elevated 1 m above the floor with two opposite open arms and two opposite closed arms (20-cm-tall walls on the closed arms) arranged at right angles. The number of entries and time spent in the closed and open arms were monitored for 10 min by ANY-Maze Video Tracking System. The maze was cleaned with 70% ethanol and water between tests.

### Novelty-suppressed feeding test

Rats were subjected to food deprivation for 48 h and then individually placed in an open-field arena (60 × 60 cm). A single pellet of the rat’s normal food chow was placed in the center of the open-field. Each animal was placed in a corner of the arena and allowed to explore the open field for up to 12 min. The latency to begin feeding and the amount of food consumed during test were recorded. The amount of time to take the first bite was recorded as latency to feed. Following the test, the rats were returned to their home cages, where food consumption was monitored for another 30 min to determine if there were any changes in appetite.

### Forced swimming

Rats were placed in a cylinder of water (temperature 24–25°C; 20 cm in diameter, 40 cm in height) for 10 min. The depth of water was set to prevent animals from touching the bottom with their hind limbs. Animal behavior was recorded by ANY-Maze Video Tracking System from the side. The following behaviors were measured: (1) latency to immobility, which was defined as floating or the least movement to maintain the head above the water; (2) time spent in struggling during the last 4 min, which was defined as strongly moving all four limbs with the front paws breaking the water surface or scratching the cylinder wall.

### Immunohistochemistry

To label newborn cells, BrdU (100 mg/kg, i.p.) was administered three times in 24-h intervals. Twenty-four hours after last BrdU injection, the animals were deeply anesthetized and transcardially perfused with 4% paraformaldehyde in 100 mM phosphate buffer, pH 7.4. Immunohistochemistry was performed on 30-μm coronal sections as previously described (Qing et al., 2008; Contestabile et al., 2013). Every sixth slice with the same reference position was mounted onto slides for staining. Immunohistochemical staining was performed on floating sections. Positive cells were counted using a 40× objective (Leica). Resulting numbers were multiplied by 6 to obtain the estimated total number of positive cells per DG of rat.

### Electrophysiology

Acute Hippocampal slices were prepared from Sprague–Dawley rats (aged 5–7 weeks). Rats were deeply anesthetized using 30% chloralic hydras (3 ml/kg, i.p.) and transcardially perfused with N-methyl-D-glucamine (NMDG) artificial cerebral spinal fluid (ACSF) prior to decapitation. NMDG ACSF contained (in mM): NMDG 93, HCl 93, KCl 2.5, NaH_2_PO_4_ 1.2, CaCl_2_ 0.5, MgSO_4_ 10, NaHCO_3_ 30, HEPES 20, Na-ascorbate 5.0, Na-pyruvate 3.0, Thiourea 2.0, NAC 12, and D-glucose 25, pH 7.3. Rat brains were rapidly dissected from the skull and placed for sectioning in ice-cold cutting solution (NMDG ACSF) bubbled with 95% O_2_ and 5% CO_2_. Coronal hippocampal slices (400 μm thickness) were sectioned from the middle third of hippocampus with a vibratome (VT1000S, Leica Microsystems, Bannockburn, IL) in cutting solution. Slices were then incubated in oxygenated HEPES ACSF for 1 h at 30°C. HEPES ACSF contained (in mM): NaCl 92, KCl 2.5, NaH_2_PO_4_ 1.2, CaCl_2_ 0.5, MgSO_4_ 10, NaHCO_3_ 30, HEPES 20, Na-ascorbate 5.0, Na-pyruvate 3.0, Thiourea 2.0, NAC 12, and 25 D-glucose, pH 7.3. Hippocampal CA1 pyramidal neurons were visualized for whole-cell patch-clamp recordings under infrared/differential interference contrast microscopy (BX51WI, Olympus, Tokyo, Japan). All recordings were conducted at room temperature (about 25^˚^C) using a Multiclamp EPC 10 amplifier (HEKA Electronics, Lambrecht/Pfalz, Germany).

For recording spontaneous excitatory postsynaptic currents (sEPSCs), the patch-pipette internal solution contained (in mM): Cs-methanesulfonate 130, MgCl_2_ 2.0, EGTA 0.5, HEPES 10, QX-314 5.0, K_2_ATP 5.0, and Na_2_GTP 0.3, pH 7.3. Filled electrodes had resistances of 3–5 MΩ. sEPSCs were pharmacologically isolated by blocking GABA receptors with bicuculline methiodide (10 μM) in standard ACSF (in mM: NaCl 120, KCl 2.5, NaH_2_PO_4_ 1.25, CaCl_2_ 2.0, MgSO_4_ 2.0, NaHCO_3_ 26, glucose 10, pH 7.3). The holding potential was −70 mV. For recording spontaneous inhibitory postsynaptic currents (sIPSCs), the external bath solution was standard ACSF with CNQX (20 μM) and AP-5 (50 μM) to block AMPA and NMDA receptors. The pipette solution contained (in mM): CsCl 140, CaCl_2_ 0.1, MgCl_2_ 2.0, HEPES 10, EGTA 0.5, and Na_2_ATP 4.0. The holding potential was −70 mV. Despite the lack of QX-314 in the intracellular solution, we seldom observed spikes in the present conditions.

Data acquisition (filtered at 3 kHz and digitized at 10 kHz) was performed with PatchMaster v2.73 software (HEKA Electronic, Lambrecht/Pfalz, Germany). sEPSCs and sIPSCs were detected automatically using Mini Analysis Program 6.0.3 (Synaptosoft Inc., Decatur, GA). Frequency and amplitude histograms were constructed using this program. The access resistance (Ra) in the present experiment is about 15 M and the change of Ra during recording is less than 10–15%, which suggests that our whole-cell recording is very stable.

### Statistical analysis

All data were analyzed with a two-way ANOVA for main effects of treatment (control or neonatal isolation), drug (saline or LiCl), and their interactions. Furthermore, to determine the influences of gender on behavioral and electrophysiological measurements, the data were analyzed with a two-way ANOVA for main effects of treatment (control or neonatal isolation), gender (male or female), and their interactions. All significant main effects and interactions were further analyzed using Turkey’s comparisons. Significance level was set at *p* < 0.05.

## Results

### Lithium rescued social deficits induced by neonatal isolation

**Figure 1:**
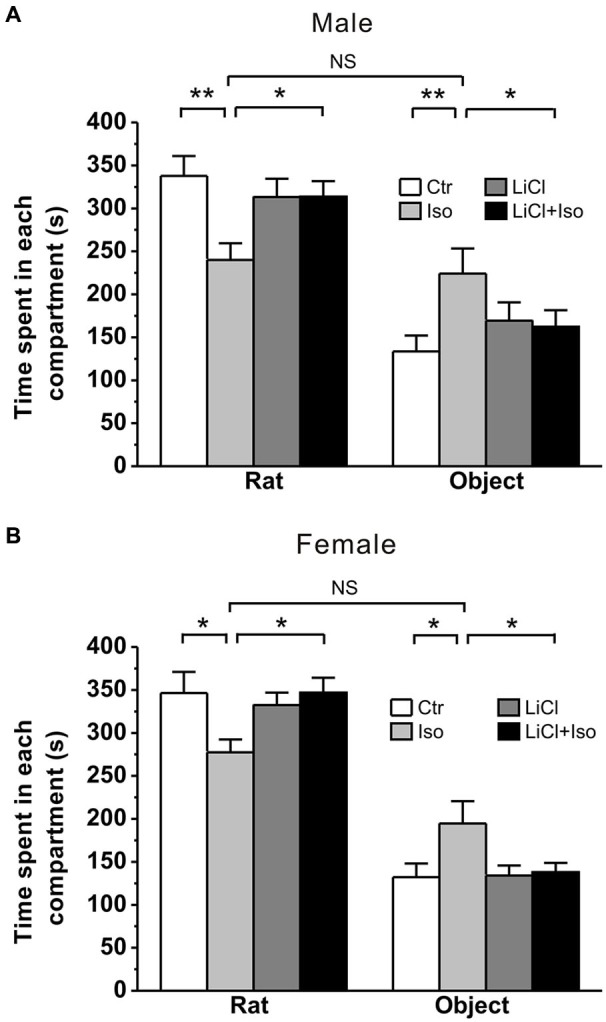
**Lithium rescues the deficit of social interaction in neonatal isolation rats**. Both male **(A)** and female **(B)** rats subjected to maternal separation for 1 h/day from PND 1–9 exhibit remarkably more time in object’s compartment and less time in stranger rat’s compartment during social interaction test compared to normal reared controls. Lithium injection immediately after daily isolation recovers the impairment of social interaction behavior in both male **(A)** and female **(B)**. Two-way ANOVA analysis was used in this experiment. For isolated male rats in stranger rat’s compartment: treatment × drug, *F*_(1, 76)_ = 5.588, *P* < 0.05; treatment, *F*_(1, 76)_ = 5.515, *P* < 0.05; drug, *F*_(1, 76)_ = 1.403, *P* > 0.05. For isolated female rats in stranger rat’s compartment: treatment × drug, *F*_(1, 76)_ = 5.111, *P* < 0.05; treatment, *F*_(1, 76)_ = 5.179, *P* < 0.05; drug, *F*_(1, 76)_ = 2.236, *P* > 0.05. For isolated male rats in object’s compartment: treatment × drug, *F*_(1, 76)_ = 4.374, *P* < 0.05; treatment, *F*_(1, 76)_ = 4.218, *P* < 0.05; drug, *F*_(1, 76)_ = 0.307, *P* > 0.05. For isolated female rats in object’s compartment: treatment × drug, *F*_(1, 76)_ = 3.984, *P* < 0.05; treatment, *F*_(1, 76)_ = 5.161, *P* < 0.05; drug, *F*_(1, 76)_ = 3.473, *P* > 0.05. Data show mean ± SEM (*p < 0.05; **p < 0.01).

Early life events, such as neonatal isolation, may lead to long-term behavioral alterations. To determine whether neonatal isolation can produce autistic-like behaviors, we firstly tested social interaction, a core symptom of autism, in young adult rats that were subjected to maternal separation during PND 1–9. The result showed that during a three-chambered social interaction test performed at PND 42–56, the normally reared male controls (Ctr: *n* = 20) spent 337.8 ± 23.2 s and 133.5 ± 18.5 s in stranger rat and object compartment, respectively (Figure 1A). However, in comparison with the controls, the isolated male rats (Iso: *n* = 22) spent significantly less time in stranger rat-containing compartment (240.1 ± 19.4 s, *p* < 0.001 vs. Ctr; Figure 1A) and more time in object-containing compartment (224.1 ± 29.3 s, *p* = 0.005 vs. Ctr; Figure 1A). Similarly, in comparison with normally reared controls (*n* = 18), isolated female animals (*n* = 16) also spent significantly less time in stranger rat-containing compartment (Ctr: 346.4 ± 26.0 s; Iso: 277.4 ± 16.5 s; *p* = 0.022; Figure 1B) and more time in object compartment (Ctr: 132.1 ± 16.7 s; Iso: 194.7 ± 28.2 s; *p* = 0.010; Figure 1B). These results are consistent with the idea that neonatal isolation may impair social interaction behavior. Notably, we found that daily LiCl (2mmol/kg, i.p.) treatment immediately after isolation, while having no effects on its own in both male (LiCl: *n* = 15, 313.3 ± 21.2 s in stranger rat-containing compartment, *p* = 0.535 vs. Ctr; 169.3 ± 21.4 s in object compartment, *p* = 0.411 vs. Ctr; Figure 1A) and female rats (LiCl: *n* = 26, 332.3 ± 14.8 s in stranger rat-containing compartment, *p* = 0.723 vs. Ctr; 134.0 ± 12.0 s in object compartment, *p* = 0.852 vs. Ctr; Figure 1B), succeeded in restoring the social interaction behavior to control levels in both isolated male (LiCl+Iso: *n* = 23, 313.7 ± 18.0 s in stranger rat-containing compartment, *p* = 0.591 vs. Ctr, *p* = 0.885 vs. LiCl, and *p* = 0.003 vs. Iso; 162.4 ± 19.1 s in object compartment, *p* = 0.363 vs. Ctr, *p* = 0.994 vs. LiCl, *p* = 0.045 vs. Iso; Figure 1A) and female rats (Licl+Iso: *n* = 20; 346.8 ± 17.3 s in stranger rat compartment, *p* = 0.671 vs. Ctr, *p* = 0.408 vs. LiCl, *p* = 0.016 vs. Iso; 138.0 ± 10.7 s in object compartment, *p* = 0.920 vs. Ctr, *p* = 0.934 vs. LiCl, *p* = 0.015 vs. Iso; Figure 1B). These results indicate that neonatal isolation produces autistic-like social deficits, and chronic lithium administration can overcome it.

### Lithium rescued abnormal repetitive self-grooming behavior induced by neonatal isolation

**Figure 2:**
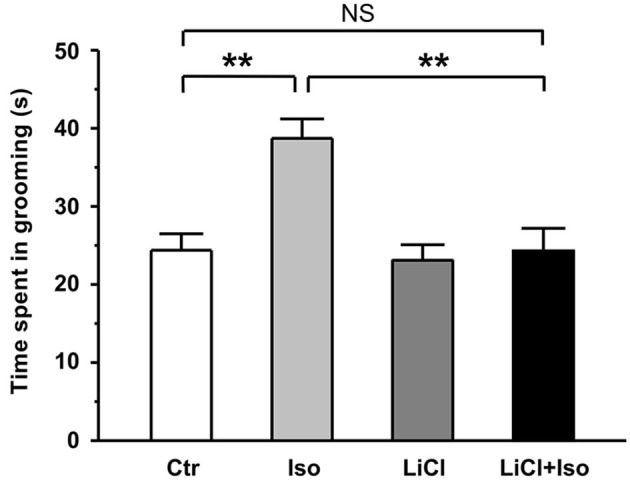
**Lithium reduces repetitive self-grooming behavior in neonatal isolation rats**. The time spent in grooming is strongly increased in isolated rats during test, and lithium treatment restores the excessive grooming behavior to normal level. Two-way ANOVA analysis was used in this experiment. For repetitive self-grooming time: treatment × drug, *F*_(1, 135)_ = 4.214, *P* < 0.05; treatment, *F*_(1, 135)_ = 6.008, *P* < 0.05; drug, *F*_(1, 135)_ = 6.191, *P* < 0.05. Data show mean ± SEM (*p < 0.05; **p < 0.01).

In addition to social interaction deficits, it is well documented that self-grooming behavior in animals may mimic several aspect of repetitive behaviors in autistic children (Lewis et al., 2007; Silverman et al., 2010; Bishop and Lahvis, 2011). Thus, we next examined whether neonatal isolation resulted in an abnormal self-grooming behavior. Since there was no significant difference in self-grooming behavioral test between the male and female rats (treatment × gender: *F*_(1, 62)_ = 3.347, *P* > 0.05; treatment: *F*_(1, 62)_ = 6.655, *P* < 0.05; gender: *F*_(1, 62)_ = 1.074, *P* > 0.05), we combined the male and female animals together for statistical analysis. We here found that animals subjected to neonatal isolation displayed significantly higher self-grooming behavior relative to control (Ctr: *n* = 30, 24.4 ± 2.1 s; Iso: *n* = 36, 38.7 ± 4.5 s; *p* = 0.007; Figure 2). Importantly, lithium treatment could completely restored the excessive self-grooming behavior to the control level (LiCl: *n* = 35, 23.1 ± 2.0 s, *p* = 0.754 vs. Ctr; LiCl+Iso: *n* = 38, 24.3 ± 2.9 s, *p* = 0.966 vs. Ctr, *p* = 0.773 vs. LiCl, *p* < 0.001 vs. Iso; Figure 2). These results suggest that neonatal isolation may produce autistic-like repetitive behavior, and lithium can reduce the excessive self-grooming behavior.

### Lithium reduced anxiety and depressive behaviors induced by neonatal isolation

Recent studies have shown that increased emotional abnormalities, such as depression and anxiety, are frequently observed in children with ASDs (Leyfer et al., 2006; Simonoff et al., 2008). Thus, we next introduced different behavioral tests, including elevated plus maze, novelty-suppressed feeding and forced swimming, to determine the influences of neonatal isolation on anxiety-/depressive-like behaviors. Since there were no significant difference in following behavioral tests between the male and female rats (For elevated plus maze: treatment × gender, *F*_(1, 41)_ = 0.092, *P* > 0.05; treatment, *F*_(1, 41)_ = 4.815, *P* < 0.05; gender, *F*_(1, 41)_ = 2.351, *P* > 0.05. For novelty-suppressed feeding: treatment × gender, *F*_(1, 35)_ = 0.001, *P* > 0.05; treatment, *F*_(1, 35)_ = 5.755, *P* < 0.05; gender, *F*_(1, 35)_ = 1.492, *P* > 0.05. For forced swimming: treatment × gender, *F*_(1, 40)_ = 1.145, *P* > 0.05; treatment, *F*_(1, 40)_ = 14.273, *P* < 0.05; gender, *F*_(1, 40)_ = 1.109, *P* > 0.05.), we combined the male and female animals together for statistical analysis.

**Figure 3:**
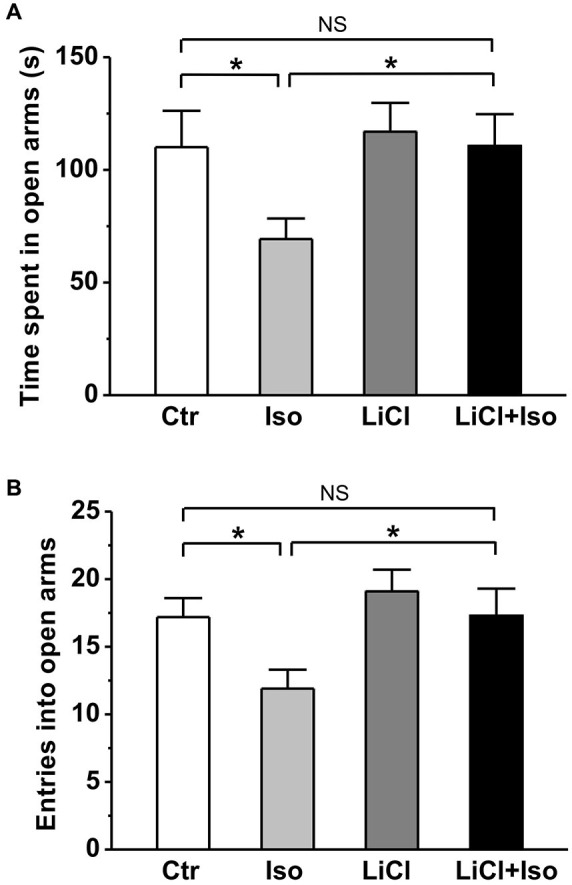
**Lithium reduces neonatal isolation-induced anxiety-like behavior in the elevated plus maze test**. Both time spent in open arms **(A)** and the number of entries into open arms **(B)** are dramatically reduced in isolated rats during test, and lithium treatment rescues the impairment. Two-way ANOVA analysis was used in this experiment. For time spent in open arms: treatment × drug, *F*_(1, 96)_ = 1.354, *P* > 0.05; treatment, *F*_(1, 96)_ = 4.497, *P* < 0.05; drug, *F*_(1, 96)_ = 4.651, *P* < 0.05. For entries into open arms: treatment × drug, *F*_(1, 96)_ = 0.886, *P* > 0.05; treatment, *F*_(1, 96)_ = 5.134, *P* < 0.05; drug, *F*_(1, 96)_ = 5.389, *P* < 0.05. Data show mean ± SEM (*p < 0.05; **p < 0.01).

Similar to previous reports in patient with autism, rats subjected to neonatal isolation exhibited increased anxiety. Compared to controls, both the time spent in open arms (Ctr: *n* = 22, 110.1 ± 16.1 s; Iso: *n* = 23, 69.3 ± 9.1 s; *p* = 0.039; Figure 3A) and the number of entry into open arms (Ctr: 17.2 ± 1.4; Iso: 11.9 ± 1.4; *p* = 0.019; Figure 3B) were significantly reduced in isolated rats during elevated plus maze test. Similar to social interaction and self-grooming test, lithium treatment was able to restore the time in open arms (LiCl: *n* = 30, 116.9 ± 12.8 s, *p* = 0.904 vs. Ctr; LiCl+Iso: *n* = 25, 110.6 ± 14.1 s, *p* = 0.999 vs. Ctr, *p* = 0.901 vs. LiCl, *p* = 0.017 vs. Iso; Figure 3A) and the number of entry into the open arms (LiCl: 19.1 ± 1.6, *p* = 0.339 vs. Ctr; LiCl+Iso: 17.3 ± 2.0, *p* = 0.987 vs. Ctr, *p* = 0.3317 vs. LiCl, *p* = 0.015 vs. Iso; Figure 3B) to control levels, indicating chronic lithium administration overcome neonatal isolation-induced anxiety.

**Figure 4:**
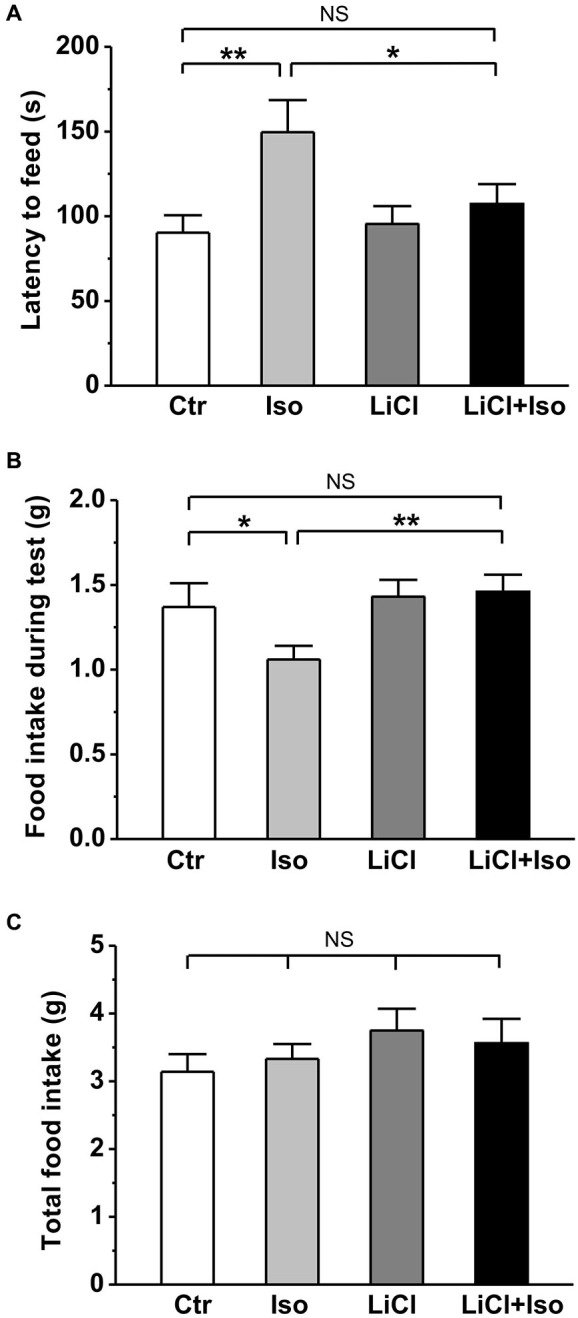
**Lithium reduces neonatal isolation-induced anxiety-like behavior in the novelty-suppressed feeding test**. The feeding latency **(A)** is significantly increased; while the amount of food intake **(B)** is significantly reduced in isolated rats compared to control. Lithium treatment reinstates this behavior. **(C)** No differences are observed in total food consumption including during test and in home cage among these groups. Two-way ANOVA analysis was used in this experiment. For latency to intake: treatment × drug, *F*_(1, 74)_ = 3.064, *P* > 0.05; treatment, *F*_(1, 74)_ = 6.901, *P* < 0.05; drug, *F*_(1, 74)_ = 1.900, *P* > 0.05. For food intake during test: treatment × drug, *F*_(1, 74)_ = 2.608, *P* > 0.05; treatment, *F*_(1, 74)_ = 5.022, *P* < 0.05; drug, *F*_(1, 74)_ = 1.946, *P* > 0.05. For total food intake: treatment × drug, *F*_(1, 74)_ = 0.405, *P* > 0.05; treatment, *F*_(1, 74)_ = 0.001, *P* > 0.05; drug, *F*_(1, 74)_ = 1.941, *P* > 0.05. Data show mean ± SEM (*p < 0.05; **p < 0.01).

In order to further evaluate the effect of neonatal isolation on anxiety, we introduced another behavioral model of anxiety under laboratory conditions, the novelty-suppressed feeding test. The result showed that neonatal isolation significantly increased the latency to feeding (Ctr: *n* = 18, 90.3 ± 10.3 s; Iso: *n* = 20, 149.6 ± 18.9; *p* = 0.001; Figure 4A), and reduced the amount of food consumed (Ctr: 1.37 ± 0.14 g; Iso: 1.06 ± 0.08 g; *p* = 0.029; Figure 4B) during test. Consistent with our prediction, lithium treatment overcame the influence of neonatal isolation on novelty-suppressed feeding behavior (LiCl+Iso: *n* = 18, 107.2 ± 11.7 s for latency to feeding, *p* = 0.752 vs. Ctr, *p* = 0.534 vs. LiCl, *p* = 0.014 vs. Iso; 1.46 ± 0.10 g for food consumption, *p* = 0.245 vs. Ctr, *p* = 0.952 vs. LiCl, *p* = 0.007 vs. Iso; Figures 4A,B), while lithium treatment alone has no effects on basal feeding behaviors (LiCl: *n* = 22, 95.4 ± 10.6 s for latency to feeding, *p* = 0.771 vs. Ctr; 1.43 ± 0.10 g for food consumption, *p* = 0.656 vs. Ctr; Figures 4A,B). Nonetheless, there was no difference among these groups in the total food consumption (Ctr: *n* = 18, 3.14 ± 0.26 g; Iso: *n* = 20, 3.33 ± 0.22 g; LiCl: *n* = 18, 3.75 ± 0.32 g; LiCl+Iso: *n* = 20, 3.56 ± 0.36 g; Figure 4C), including food intake during test and in the home cage, suggesting that neonatal isolation affected animal’s anxiety rather than appetite.

**Figure 5:**
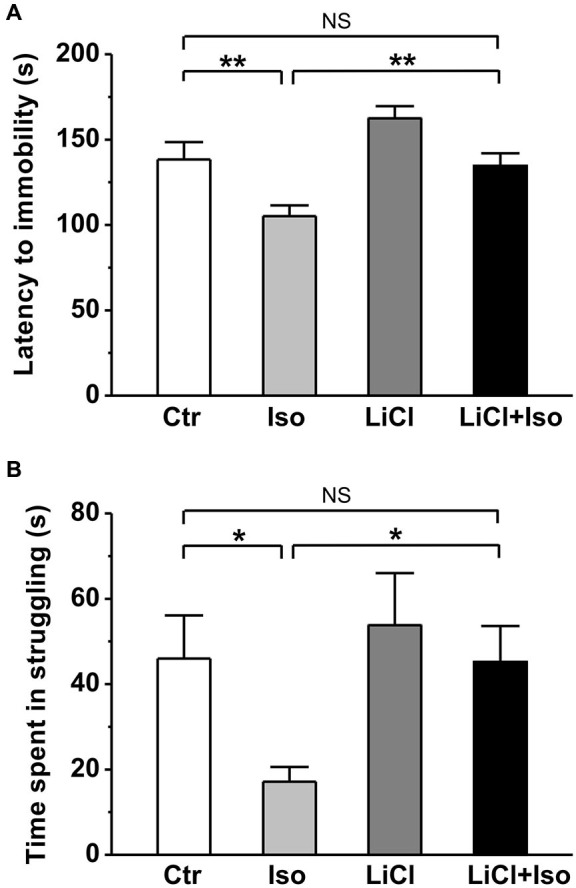
**Lithium reduces depressive-like behavior in neonatal isolation rats**. In forced swimming paradigm, both latency to immobility **(A)** and struggling time during the last 4 min **(B)** are significantly reduced in isolated rats compared to control, and lithium treatment recovers the impairment. Two-way ANOVA analysis was used in this experiment. For latency to immobility: treatment × drug, *F*_(1, 95)_ = 9.779 *P* < 0.05; treatment, *F*_(1, 95)_ = 19.003, *P* < 0.05; drug, *F*_(1, 95)_ = 3.473, *P* > 0.05. For time in struggling: treatment × drug, *F*_(1, 95)_ = 1.178, *P* > 0.05; treatment, *F*_(1, 95)_ = 3.968, *P* < 0.05; drug, *F*_(1, 95)_ = 3.648, *P* > 0.05. Data show mean ± SEM (*p < 0.05; **p < 0.01).

Next, using forced swimming paradigm, we tested the influence of neonatal isolation on depressive-like behavior, another emotional comorbidity in patient with autism. The results showed that neonatal isolation significantly reduced both latency to immobility (Ctr: *n* = 20, 144.0 ± 10.3 s; Iso: *n* = 24, 105.2 ± 6.3; *p* < 0.001; Figure 5A) and duration of struggling (Ctr: 46.0 ± 10.1 s; Iso: 24.2 ± 7.3 s; *p* = 0.042; Figure 5B) during the forced swimming test, indicating increased depression in the isolated group. In agreement with an anti-depressive effect of lithium, we found that while having little effects on its own (LiCl: *n* = 28, 162.5 ± 7.1 s for latency to immobility, *p* = 0.087 vs. Ctr; 53.8 ± 12.2 s for struggling time; *p* = 0.765 vs. Ctr), lithium treatment abolished the influence of neonatal isolation on depressive behavior (LiCl+Iso: *n* = 26, 134.6 ± 7.4 s for latency to immobility, *p* = 0.546 vs. Ctr, *p* = 0.082 vs. LiCl, *p* = 0.005 vs. Iso; 53.8 ± 12.2 s for struggling time, *p* = 0.865 vs. Ctr, *p* = 0.533 vs. LiCl, *p* = 0.031 vs. Iso; Figures 5A,B).

Taken together, these results indicate that neonatal isolation increases animal’s anxiety and depression, and chronic lithium administration can overcome these abnormal emotional alterations.

### Lithium treatment prevented neonatal isolation-induced decrease in adult hippocampal neurogenesis

**Figure 6:**
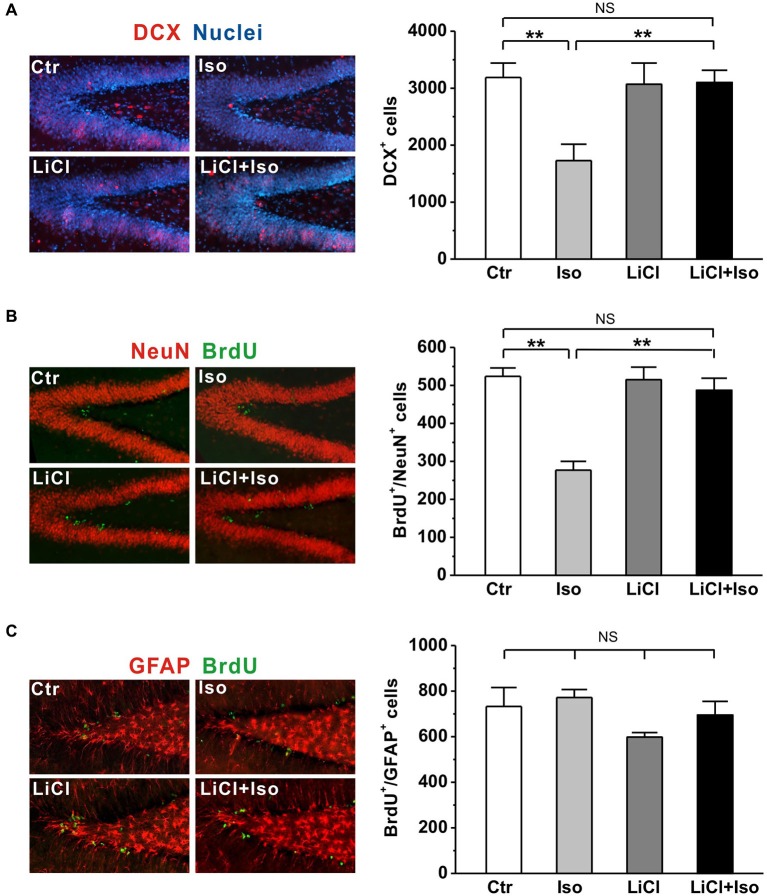
**Lithium rescues adult hippocampal neurogenesis in neonatal isolation rats. (A)** Neonatal isolation reduces immunoreactivity (left panel) and number of DCX^+^ newborn neurons (right bar) in the DG, and lithium restores the number of newborn neurons to control level. **(B)** Neonatal isolation reduces immunoreactivity (left panel) and number of newborn neuronal phenotype cells (right bar) in the DG, and lithium restores the number of newborn neurons to control level. **(C)** Neither neonatal isolation nor lithium has any effect on immunoreactivity (left panel) and number of newborn astrocytic phenotype cells (right bar) in the DG. Two-way ANOVA analysis was used in this experiment. For DCX^+^ newborn neurons: treatment × drug, *F*_(1, 20)_ = 6.708, *P* < 0.05; treatment, *F*_(1, 20)_ = 6.179, *P* < 0.05; drug, *F*_(1, 20)_ = 4.737, *P* < 0.05. For BrdU^+^/NeuN^+^: treatment × drug, *F*_(1, 20)_ = 15.314, *P* < 0.05; treatment, *F*_(1, 20)_ = 24.147, *P* < 0.05; drug, *F*_(1, 20)_ = 12.900, *P* < 0.05. For BrdU^+^/GFAP^+^: treatment × drug, *F*_(1, 20)_ = 0.280, *P* > 0.05; treatment, *F*_(1, 20)_ = 1.541, *P* > 0.05; drug, *F*_(1, 20)_ = 3.744, *P* > 0.05. Data show mean ± SEM (*p < 0.05; **p < 0.01).

It has been reported that neonatal isolation impairs neurogenesis in the DG (Rizzi et al., 2007) and reduced neurogenesis may contribute to neuropathogensis of ASDs (Wegiel et al., 2010). Thus, we next tested whether the neurogenesis impairment was reproduced in our animals and if so, whether it could be prevented by chronic lithium treatments. To observe newborn neurons, we used the early neuronal marker doublecortin (DCX; Brown et al., 2003) to stain the immature neurons. We observed that the number of DCX-expressing immature neurons was dramatically reduced in the DG of rats subjected to neonatal isolation (Ctr: *n* = 6, 3189 ± 251; Iso: *n* = 6, 1728 ± 290; *p* < 0.001; Figure 6A); while lithium treatment fully restored the number of DCX-positive neurons to control levels (LiCl: *n* = 6, 3070 ± 372; LiCl+Iso: *n* = 6, 3100 ± 215, *p* = 0.846 vs. Ctr, *p* = 0.595 vs. LiCl, *p* = 0.001 vs. Iso; Figure 6A). To further confirm the impairment of NPC proliferation in the DG of isolated rats, we treated animals with BrdU daily from PND 42–44, and sacrificed them at PND 45. Since different phenotypes of the proliferated cells can be labeled by BrdU, we used immunofluorescent double-labelings of brain sections with BrdU and either a neuronal (NeuN) or an astrocyte (GFAP) marker. We found that neonatal isolation significantly reduced the number of BrdU-positive cells with neuronal phenotype (Ctr: *n* = 6, 524 ± 22; Iso: *n* = 6, 277 ± 23; *p* < 0.001; Figure 6B), rather than astrocytic phenotype (Ctr: *n* = 6, 733 ± 83; Iso: *n* = 6, 772 ± 35; *p* = 0.650; Figure 6C). Notably, lithium treatment, while having no effect on its own (LiCl: *n* = 6, 515 ± 33; *p* = 0.606 vs. Ctr; Figure 6B), promoted the proliferation of NPCs in the DG of isolated rats, thereby restoring the number of newborn neurons to physiological levels (LiCl+Iso: *n* = 6, 487 ± 32, *p* = 0.413 vs. Ctr, *p* = 0.758 vs. LiCl, *p* < 0.001 vs. Iso; Figure 6B). These results indicate that neonatal isolation impairs adult hippocampal neurogenesis, reducing the number of newly generated neurons, and chronic lithium administration can prevent this negative effect on neurogenesis.

### Lithium restored the neonatal isolation-enhanced inhibitory synaptic transmission

**Figure 7:**
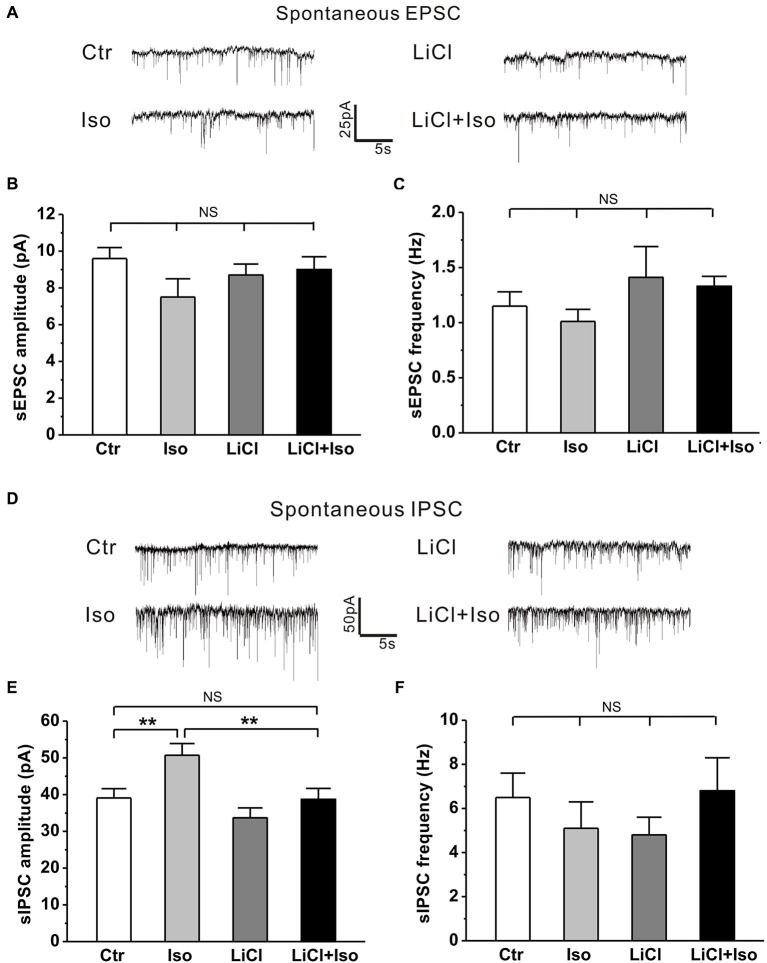
**Lithium readjusts the balance between excitatory and inhibitory synaptic activities in neonatal isolation rats**. (**A–C)** Neither neonatal isolation nor lithium has any effect on sEPSC amplitude **(B)** or frequency **(C)**, and corresponding representative traces are shown in graph **(A)**. For sEPSC amplitude: treatment × drug, *F*_(1, 54)_ = 1.981, *P* > 0.05; treatment, *F*_(1, 54)_ = 2.066, *P* > 0.05; drug, *F*_(1, 54)_ = 0.352, *P* > 0.05. For sEPSC frequency: treatment × drug, *F*_(1, 54)_ = 0.485, *P* > 0.05; treatment, *F*_(1, 54)_ = 1.608, *P* > 0.05; drug, *F*_(1, 54)_ = 0.031, *P* > 0.05. **(D–F)** Neonatal isolation dramatically enhances sIPSC amplitude **(E)**, but not frequency **(F)**; while lithium recues the abnormal enhancement of sIPSC amplitude, and corresponding representative traces are shown in graph **(D)**. For sIPSC amplitude: treatment × drug, *F*_(1, 47)_ = 1.253, *P* > 0.05; treatment, *F*_(1, 47)_ = 8.143, *P* < 0.05; drug, *F*_(1, 47)_ = 8.797, *P* < 0.05. For sIPSC frequency: treatment × drug, *F*_(1, 47)_ = 2.210, *P* > 0.05; treatment, *F*_(1, 47)_ = 0.057, *P* > 0.05; drug, *F*_(1, 47)_ = 0.001, *P* > 0.05. Data show mean ± SEM (*p < 0.05; **p < 0.01).

Recent studies have revealed that the synaptic function is altered and manifested as an excitation-inhibition imbalance due to an increased synaptic inhibition in genetic animal models of autism (Tabuchi et al., 2007; Pizzarelli and Cherubini, 2013). However, whether neonatal isolation has a similar synaptic excitation-inhibition imbalance, thereby contributing to the pathogenesis of the observed autistic phenotypes, remains undetermined. Therefore, we next examined both inhibitory and excitatory synaptic transmission in CA1 pyramidal neurons from hippocampal slices. Similar to aforementioned behavioral results, there was no significant difference in sIPSC amplitude between the male and female rats (treatment × gender: *F*_(1, 26)_ = 1.646, *P* > 0.05; treatment: *F*_(1, 26)_ = 7.919, *P* < 0.05; gender: *F*_(1, 26)_ = 0.027, *P* > 0.05), we combined the male and female animals together for statistical analysis. The results showed that neonatal isolation had no effects on either sEPSC amplitude (Ctr: *n* = 18, 9.6 ± 0.6 pA; Iso: *n* = 14, 7.5 ± 1.0 pA, *p* = 0.233; Figures 7A,B) or sEPSC frequency (Ctr: *n* = 18, 1.2 ± 0.1 Hz; Iso: *n* = 14, 1.0 ± 0.1 Hz; *p* = 0.325; Figures 7A,C), but significantly enhanced sIPSC amplitude (Ctr: *n* = 18, 39.1 ± 2.5 pA; Iso: *n* = 12, 50.7 ± 3.2 pA; *p* = 0.007; Figures 7D,E), without affecting sIPSC frequency (Ctr: *n* = 18, 6.5 ± 1.1 Hz; Iso: *n* = 12, 5.1 ± 1.2 Hz; *p* = 0.214; Figures 7D,F). Notably, lithium treatment, while having no effect on either sEPSC amplitude (LiCl: *n* = 13, 8.7 ± 0.6 pA, *p* = 0.783 vs. Ctr; Figures 7A,B) and frequency (Licl: *n* = 13, 1.4 ± 0.3 Hz, *p* = 0.891 vs. Ctr; Figures 7A,C) or sIPSC amplitude (LiCl: *n* = 11, 33.7 ± 2.7 pA, *p* = 0.123 vs. Ctr; Figures 7D,E) and frequency (LiCl: *n* = 11, 4.8 ± 0.8 Hz, *p* = 0.325 vs. Ctr; Figures 7D,F) on its own, succeeded in restoring the sIPSC amplitude in isolated animals to control levels (LiCl+Iso: *n* = 10, 38.7 ± 3.0 pA, *p* = 0.922 vs. Ctr, *p* = 0.225 vs. LiCl, *p* = 0.006 vs. Iso; Figures 6D and 7E). Altogether, these results indicate that neonatal isolation increases inhibitory synaptic transmission without affecting excitatory synapses, and lithium treatment succeeds in reinstating it to physiological level.

## Discussion

The main findings of the present study are that young adult rats that were subjected to neonatal isolation from PND 1–9 display behavioral phenotypes strikingly similar to some of the behavioral hallmark features of autistic patients, such as social deficits, repetitive self-grooming and anxiety-/depressive-like behaviors. These behavioral abnormalities are associated with impaired hippocampal neurogenesis and abnormally enhanced inhibitory synaptic transmission. More importantly, lithium administration immediately after isolation is effective to overcome neonatal isolation-induced changes in behaviors, neurogenesis and synaptic transmission. These findings indicate that neonatal isolation may produce autistic-like behaviors, and lithium may be a potentially effective therapy for autism.

### Neonatal isolation produces autistic-like behaviors

Animal models of human diseases, especially some complex disorders, such as autism, are likely to suffer from many shortcomings. Although more and more evidence supports that genetic factor is the predominant cause of the ASD, hundreds of genes are now associated with ASD (Abrahams and Geschwind, 2008; State and Levitt, 2011; Devlin and Scherer, 2012), suggesting that there are diverse genetic risk factors for autism. Therefore, it is difficult to generate an animal model via a single genetic variant to mimic autism syndromes in patients. Neonatal isolation has been widely accepted as an animal model to study the long-term behavioral alterations, including cognitive deficit, lower sensitivity to pain, impaired sensorimotor gating and increased anxiety/depression (Kosten et al., 2007; Weaver et al., 2007; Marco et al., 2013), which are common comorbidities in children with autism (Perry et al., 2007; Mazefsky et al., 2010; Strang et al., 2012). We therefore proposed that neonatal isolation may be a simple animal model of autistic-like behaviors. Indeed, in the present study, we provided several lines of evidence that confirms many of the previous behavioral findings in neonatal isolation model, and suggests that this animal model may be a reproducible animal model for ASD research. Firstly, we demonstrated that neonatal isolation can reproducibly exhibit increased anxiety (Figures 3 and 4) and depression (Figure 5). Secondly, our results also showed that neonatal isolation resulted in an apparent deficit in social interactions (Figure 1) and excessive self-grooming behavior (Figure 2), which are hallmark features of autism (American Psychiatric Association, 1994). Finally, consistent with a previous report (Rizzi et al., 2007), we also found that neonatal isolation dramatically impaired adult neurogenesis (Figure 6), which may contribute to neuropathogensis of ASDs (Wegiel et al., 2010). Altogether, our results, along with evidence accumulated from previous studies using neonatal isolation rat model, indicate that this model has striking similarities with the human autistic phenotype and may be a valid animal model of autistic-like behaviors.

However, how neonatal isolation produces autistic-like behavioral phenotype remains unknown. Consistent with previous studies (Rizzi et al., 2007; Raceková et al., 2009), we here reported that neonatal isolation resulted in a dramatic decrease in the number of newborn neurons in the DG (Figure 6). Thus, a plausible conjecture is that neonatal isolation impaired adult hippocampal neurogenesis, which may contribute to the observed excitation-inhibition imbalance observed in the present work (Figure 7), leading to the formation of autistic-like behaviors. Notably, besides hippocampal alterations, neonatal isolation can disrupt dendritic morphology of neurons and decrease the stability of mushroom spines in other brain regions, such as prefrontal cortex and somatosensory cortex (Takatsuru et al., 2009; Monroy et al., 2010), which may also contribute to the formation of autistic-like behaviors. In addition, recent studies proposed that maternal separation was associated with DNA methylation in adult rats (Lewis et al., 2013; Marco et al., 2013), suggesting that neonatal isolation may produce autistic-like behaviors through the mechanism of epigenetic regulation. So far, although it is hard to obtain direct causality between autism and neurogenesis or epigenetic change, it provides a direction for future research.

### Lithium: a potential therapy for autism during development

In addition to the well-documented mood-stabilizing effects of lithium in manic-depressive patients, increasing evidence from *in vitro* and *in vivo* studies have implicated that lithium can be used in the treatment of acute brain injuries (Nonaka and Chuang, 1998; Ren et al., 2003) and central nervous system diseases, such as Alzheimer’s disease (De Ferrari et al., 2003), Huntington’s disease (Senatorov et al., 2004), fragile X (Guo et al., 2011) and Down syndrome (Bianchi et al., 2010; Contestabile et al., 2013). Based on these advances, we speculate that lithium may be an effective drug to treat autism. Indeed, the present study showed that lithium treatment fully reversed autistic features, such as social interaction deficit (Figure 1), excessive repetitive self-grooming behavior (Figure 2), increased anxiety (Figures 3 and 4) and depression (Figure 5) in neonatal isolation animal model. In addition, consistent with previous findings in other central nervous system disorders (De Ferrari et al., 2003; Senatorov et al., 2004; Bianchi et al., 2010; Contestabile et al., 2013), lithium treatment restored the adult hippocampal neurogenesis to the physiological level in isolated animals (Figure 6). The exact underlying molecular mechanism remains to be determined, but may be at least in part due to the inhibition effects of lithium on glycogen synthase kinase-3β (GSK-3β), as it is a key event in its therapeutic roles in several other neuropsychiatric disorders (Beaulieu et al., 2004; O’Brien et al., 2004; Silva et al., 2008). As a competitive inhibitor of magnesium, lithium can directly inhibit Mg^2+^-ATP-dependent catalytic activity of GSK-3β (Ryves and Harwood, 2001). In addition, lithium also can increase Ser9 phosphorylation of GSK-3β (Zhang et al., 2003), thereby inhibiting GSK-3β activity (Jope, 2003). Thus, future experiments examining GSK-3β activity in neonatal-isolated animals with or without lithium treatment will help determine whether lithium’s therapeutic effect in neonatal isolation model of autism can be attributed to its inhibition of GSK-3β. Furthermore, recent study shows that lithium treatment can restore the decreased neuropeptide Y induced by maternal deprivation in the hippocampus and striatum (Husum and Mathe, 2002). Since neuropeptide Y has been shown to promote proliferation of postnatal neuronal precursor cells in rats (Hansel et al., 2001), there is another possibility that lithium treatment restored the adult hippocampal neurogenesis in animals subjected to neonatal isolation via increasing neuropeptide Y in the hippocampus. It is, however, interesting to note that no elevated neurogenesis was observed after lithium treatment in adult control rats in the current study (Figure 6). One possible explanation is that nine-day administration of lithium Kehoe the increase of neurogenesis *per se*, though it may be enough to rescue the impairment of neurogenesis induced by neonatal isolation. This hypothesis is supported by some previous reports. For example, Son et al. (2003) found short-term lithium treatment succeeded in enhancing hippocampal long-term potentiation similar to those found in long-term lithium treatment, but it could not increase neurogenesis.

Alternatively, lithium may modulate neurotransmitters and likely re-adjust balances between excitatory and inhibitory activities as previously proposed (Jope, 1999). Previous reports have shown that dysfunction of serotoninergic neurotransmission in central nervous system is involved in depression, anxiety and memory disorders. For example, increased level of brain 5-HT enhances memory (Haider et al., 2006), whereas decreased level of brain 5-HT impairs cognitive performance (Porter et al., 2003). Thus, lithium treatment may ameliorate autistic-like behaviors in the present study via influencing brain 5-HT metabolism (Perveen et al., 2013). In addition, similar to previous findings in genetic animal model of autism (Tabuchi et al., 2007; Pizzarelli and Cherubini, 2013), we revealed here that neonatal isolation increases sIPSC without changing sEPSC, which results in an increase in ratio of inhibitory/excitatory synaptic activities, while lithium treatment successfully restores the physiological balance between excitatory and inhibitory activities (Figure 7). So far, although we cannot conclude the exact effect of neonatal isolation or lithium on the miniature synaptic events that are included in the spontaneous synaptic events, the results of sEPSC and sIPSC are sufficient to reflect presynaptic or postsynaptic changes of synaptic function of neural network. Thus, our results not only validate the hypothesis that excitatory/inhibitory balance may be compromised in autism, but also indicate that it may be possible to ameliorate autistic-like behavioral abnormalities by regaining the balance between excitation and inhibition.

## Conclusion

Overall, our study shows that neonatal isolation results in autistic-like synaptic and behavioral phenotypes and lithium treatment is able to ameliorate these deficits. These finding indicate that neonatal isolation may be a simple animal model of autistic-like behaviors for future research, and lithium may represent a potentially effective therapeutic drug for autism.

## Conflict of interest statement

The authors declare that the research was conducted in the absence of any commercial or financial relationships that could be construed as a potential conflict of interest.
